# Aboriginal children and family connections to primary health care whilst homeless and in high housing mobility: observations from a Nurse Practitioner-led service

**DOI:** 10.1017/S1463423621000384

**Published:** 2022-03-21

**Authors:** Nina Sivertsen, Yvonne Parry, Eileen Willis, Sally Kendall, Rhonda Marriott, Alicia Bell

**Affiliations:** 1 College of Nursing and Health Sciences, Flinders University, Adelaide, SA, Australia; 2 Professor of Community Nursing and Public Health Centre for Health Services Studies, University of Kent, Canterbury, UK; 3 PVC Aboriginal and Torres Strait Islander Leadership and Director Ngangk Yira Research Centre for Aboriginal Health and Social Equity, Murdoch University, Perth, Australia

**Keywords:** aboriginal children, child focused care, children’s health access, family homelessness, Nurse Practitioner practice, vulnerable children

## Abstract

**Aim:**

This article documents the impact of a Nurse Practitioner-led primary health service for disadvantaged children living in housing instability or homelessness. It identifies that First Nations children miss out on essential primary care, particularly immunisation, but have less severe health conditions than non-First Nations children living in housing insecurity.

**Background:**

Health services for homeless populations focus on the 11% of rough sleepers, little is done for the 22% of children in Australia living in housing instability; many of whom are from First Nations families. Little is known of the health status of these children or their connections to appropriate primary health care.

**Methods:**

This research implemented an innovative model of extended health care delivery, embedding a Nurse Practitioner in a homeless service to work with families providing health assessments and referrals, using clinically validated assessment tools. This article reports on proof of concept findings on the service that measured immunisation rates, developmental, medical, dental and mental health needs of children, particularly First Nations children, using a three-point severity level scale with Level 3 being the most severe and in need of immediate referral to a specialist medical service.

**Findings:**

Forty-three children were referred by the service to the Nurse Practitioner over a 6-month period, with nine identifying as First Nations children. Differences in severity levels between First Nations/non-First Nations children were Level 1, First Nations/non-First Nations 0/15%; Level 2, 10/17%; and Level 3, 45/29%. Forty-five percent of First Nations children had no health problems, as compared to 29% on non-First Nations children. Immunisation rates were low for both cohorts. No First Nations child was immunised and only 9% of the non-First Nations children. While numbers for both cohorts are too low for valid statistical analysis, the lower levels of severity for First Nations children suggest stronger extended family support and the positive impact of cultural norms of reciprocity.

## Introduction/background

The Department of Health, Action Plan for Children and Young People states 22% of all Australian children live in housing instability (Australian Government, Department of Health, 2019). Other research states one in six children in Australia live in disadvantage or are marginalised. Australia has 1.1 million children living in disadvantage (Davidson *et al.*, 2018; The Smith Family, 2019). Internationally, current health service delivery models do not meet the needs of children living in housing instability (Pennsylvania Chapter of the American Academy of Paediatrics, 2014; Lau *et al.*, 2016; Parry *et al.*, 2016; Long *et al.*, 2018; Department of Health, 2018). Homelessness service delivery by social workers and community staff does not include comprehensive paediatric health assessments or consistently track the monitoring of referrals to primary health care (Parry *et al.*, 2016). Research also shows children who experience housing instability experience a snowballing effect of multiple and cumulative risk factors and traumas that often escalate to serious issues like chronic homelessness, crime or hospitalisation for mental health conditions (Toumbourou *et al*., 2014; Teager *et al.*, 2019; Parry *et al.*, 2020).

Children living in housing instability have poorer access to health services and appointment compliance, increased Emergency Department (ED) utilisation, and overall poorer mental and physical health outcomes (Pungello *et al.*, 2010; Ding *et al.*, 2014; Parry *et al.*, 2016; Long *et al.*, 2018; The Smith Family, 2019; Strong Foundation Collaboration, 2019). Marginalised children, particularly those from homeless families, are an at-risk group who may suffer long-term physical and mental health impacts from disadvantage. Adverse childhood experiences incurred during times of disadvantage include trauma, domestic violence and multiple toxic stressors that negatively impact long term on neurobiological development, cognitive ability and mental health (Pungello *et al.*, 2010; Davidson *et al.*, 2018; Australian Government Department of Health, 2019; The Smith Family, 2019).

Children from disadvantaged backgrounds have higher rates of acute and chronic health conditions including otitis media, asthma, scabies, respiratory infections, gastroenteritis, anxiety and depression. In addition, children living in homeless families endure six times the rates of injury and accidents, are three times more likely to have poor health and lower rates of immunisation (Davidson *et al.*, 2018; Australian Government Department of Health, 2019; The Smith Family, 2019). Current forms of health care access do not adequately meet the needs of children in families who experience homelessness increasing the prevalence of accumulative harm and long-term deleterious health outcomes. For example, it is known that children between the ages of 0–12 years make up 25% of the homeless population in Australia (Homelessness Australia, 2014; The Smith Family, 2019); their poorer health, educational and social outcomes are partly explained by their disconnection from the usual health and service provision that optimises child development and mitigates adverse childhood experiences (Warren, 2017; Long *et al.*, 2018).

Early interventions can circumvent the development and progression of lifelong ill-health and deleterious poorer mental health outcomes (Davidson *et al.*, 2018; Australian Government Department of Health, 2019; The Smith Family, 2019). The early connection of marginalised children with health services aids families saving the public both health and welfare costs (Pungello *et al.*, 2010). The numbers of children aged 0–12 years attending homeless services are increasing both in Australia and globally (Long *et al.*, 2018; Australian Government Department of Health, 2019). Access to adequate primary health care services is a major problem for homeless adults with children. Every day, two in every three homeless children (0–12 years) who require immediate accommodation from homeless services are turned away (Davidson *et al.*, 2018).

Late action in addressing preventable child developmental interventions costs Australia $15bn per year (Teager *et al.*, 2019). Effective early interventions provide opportunities for positive trajectory changes for children while saving the public billions in late intervention costs (Teager *et al.*, 2019). Ad hoc care, such as ED use, during childhood can result in inconsistent, inappropriate and detrimentally longer-term health outcomes. Emergency-only care can result in missed immunisation, physical, cognitive and behavioural developmental impairment and failure to meet milestones, due to missing educational and health appointments to address the deleterious health, education and social impacts of disadvantage (Pennsylvania Chapter of the American Academy of Paediatrics, 2014; Lau *et al.*, 2016; Long *et al.*, 2018; Department of Health, 2018). Referral uptake and appointment compliance is one of the greatest barriers for children living in homeless families (Davidson *et al.*, 2018; Australian Government, Department of Health, 2019; Smith Family, 2019). Direct referral support for children can provide improvements in physical and mental health, and behaviour (Merricka *et al.*, 2017; Valentine et al., 2020).

One of the major problems in addressing appropriate health access is the lack of data on the numbers of homeless children. For example, the children attend homelessness services with their parents and yet these services were only mandated to count the numbers of children attending with a parent in 2009 in South Australia and then in NSW in 2014 (Gallardo *et al.*, 2020; Australian Institute of Health and Welfare, 2019; Australian Government, Department of Health, 2019) making knowledge of the numbers of children exposed to this type of disadvantage only recent. Additionally, homelessness services are often the first to know when children are at risk of abuse or neglect and disconnected from other services (Parry *et al.*, 2016, 2020).

## First Nations children and homelessness

In Australia, First Nations Peoples are around 3% of the total population; however, they make up 23% of the homeless population (Homelessness Australia, 2016). This higher risk of homelessness has many factors (Homelessness Australia, 2016). These include historical and cultural characteristics that remain and are perpetuated in Australia (Memmott and Chamber, 2010). Additionally, for First Nations families the impacts of dispossession, the stolen generations and structural racism are results of the often ‘crisis management’ approach to First Nations Peoples’ homelessness (Ruttan *et al.*, 2010; Zufferey and Chong, 2015).

The lack of consultation with First Nations communities in addressing complex issues such as family homelessness often compounds their situation and makes it difficult for them to access services (Zufferey and Chong, 2015). First Nations children face many challenges when accessing mainstream services. These include unwelcoming hospital or primary care settings, lack of transport, mistrust of mainstream health care, a sense of alienation and inflexible treatment options. This can result in an overall reluctance to attend services (Shahid *et al.*, 2009). Effectively addressing health disparities between First Nations Peoples and non-First Nations Australians is long overdue. Culturally safe community care is needed that leads to increased access and trust in services (Durey *et al.*, 2016).

This article reports on the outcomes of proof of concept study of a Nurse Practitioner-led service provided for all children living in housing instability established in the Southern suburbs of Adelaide. The findings presented here outline some of the health outcomes for First Nations families in comparison to non-First Nations children. The outcomes suggest that First Nations children have better health status than non-First Nations children; we hypothesis that this is partly explained by the strong focus on extended family support by the First Nations population and the cultural norm of reciprocity (Vallesi *et al.*, 2020).

## Aim

To compare the health and welfare outcomes of First Nations and non-First Nations children living in housing instability who presented to a Nurse Practitioner-led primary service.

## Methods

We implemented an innovative model of extended health care delivery, embedding a Nurse Practitioner-led clinic in a homeless service to provide children of families living in housing insecurity with health assessments, treatment and referrals, using clinically validated assessment tools (USAID, 2013; WHO, 2016).

### Participants

The project provided services to 43 families over a 6-month period between December 2019 and August 2020. Nine of the children were First Nations children.

### The programme

The first author partnered with Inner Southern Homelessness Service (ISHS), a not for profit community provider and part of Uniting Care Wesley Bowden (UCWB), to establish and evaluate a Nurse Practitioner-led health service for families living in housing insecurity. A key component of the project was to develop referral and care access plans encompassing the health, social and educational needs of the child and assist the family to navigate the barriers to referral compliance such as transport or the cost of primary medical services. UCWB has strong links with Kornar Winmil Yunti (KWY), a First Nations family welfare service who provided cultural training for all staff. UCWB also partnered with INCOMPRO First Nations Association to deliver a kinship care programme. The UCWB’s homelessness service provides in-kind support through use of facilities and resources to book and coordinate appointments. REDACTED (inserted on acceptance) employed the Nurse Practitioner and undertook the research and evaluation on the effectiveness of this proof of concept study of the service.

Nurse Practitioners have specialist knowledge and skills, and the authority to use extended practice privileges to make informed and autonomous decisions on preventative, diagnostic and therapeutic management. Care of vulnerable populations and those living in disadvantage support the use of Nurse Practitioners as it is a cost-effective model of care (Jennings *et al.*, 2015; Woo *et al.*, 2017). In Australia, they are reimbursed through Medicare meaning the service was free; however, the assessments required for the evaluation of the study were not covered under Medicare and were separately funded. The Nurse Practitioner employed for this project was a paediatric ED Registered Nurse.

### Data collection

ISHS routinely collects data on attendance levels and referrals by staff to other organisations such as health, education and welfare services. This includes demographic data such as age, educational attendance and health service use or needs. These data are required to monitor usage of the service by families and individuals seeking assistance. There are two types of assistance provided – an intake service (emergency housing assistance) and short-term assistance (case management temporary housing).

Data collected contain the following information:

- Date of visit
- De-identified client code
- Age
- Reason for referral to Nurse Practitioner
- Previous Child and Family Health Nurse visits, ED visits and GP use
- Medical conditions and severity (three levels)
- Treatment or service provided by Nurse Practitioner
- Type of referrals arranged
- Referral support required
- Referral follow-up request
- Home visits required (due to parent with trauma history, agoraphobia or anxiety issues)
- Health outcomes
- Medicare number and status (National Health Insurance scheme)
- Blue book (a personal health record book) sighted
- Proof of immunisations
- First Nations identify
- Refugee identity.

This proof of concept study used a descriptive statistical data analysis (Mishra *et al.*, 2019). Summary measures or descriptive statistics were used to report observations (Mishra *et al.*, 2019). Descriptive statistics provide simple summaries about the sample and the measures.

### Ethical considerations

The project had ethics approval by the REDACTED (name of HREC committee inserted on acceptance), project No. 8502. All standard ethical codes and legal regulations to ensure the absence or minimisation of harm, trauma, anxiety or discomfort of human participants were followed (NHMRC, 2018).

## Findings

In presenting the finding, we outline overall access to the service and then the First Nations family specific use of the services.

### Users of the service

Information from the homeless service stated that on average 250 families or individuals attend the homeless service per month. During this proof of concept stage, 43 families were referred to the Nurse Practitioner-led service between December 2019 and August 2020. Of these 43 referrals, 21% (*n* = 9) identified as First Nations Peoples, and 7% (*n* = 3) as refugees.

### Ages, children and siblings

Overall, the average age of attendees was 7 years old, with the youngest who attended the service 7 weeks (*n* = 1) and the oldest 16 years old (*n* = 2). Of the First Nations children attending the Nurse Practitioner service, the average age was 6 years old, with the youngest 7 months old (*n* = 1), oldest 12 years old (*n* = 1) and 2 children under 1 year. Most homeless families referred also counted numerous siblings. Only four of the 43 children had no siblings, meaning that in addition to the 43 children seen at the Nurse Practitioner service, families counted more than 100 children siblings (*n* = 105). For the First Nations cohort, the number of siblings was almost 30 (*n* = 28), making the total number of First Nations children in the homeless families seen including siblings 37 (*n* = 37).

### Presenting medical conditions and severity

All children referred to the Nurse Practitioner-led service received a comprehensive and in-depth health assessment. The children presented with various severity-level medical conditions. The conditions identified were coded using a scale of severity =3 (needing a referral immediately), moderate = 2 (needing a referral but can wait for public clinic) and minor = 1 (does not need immediate referral). Overall the presentations of children to the clinic showed the high rates of of health conditions (56%) requiring medical and health intervention versus the 44% of children who were assessed to be in good health. Table 1 outlines the severity levels and immunisation rates of both First Nations children and the non-First Nations cohort.

**Table 1.** Categorisation of non-First Nations and First Nations Children severity levels

Status	Severity Level 1	Severity Level 2	Severity Level 3	No health problems	Not immunised	No severity rating
Non-First Nations *N* = 34 (79%)	5 (15%)	6 (17%)	10 (29%)	10 (29%)	30 (88%)	3 (10%)
First Nations children *N* = 9 (21%)	0 (0%)	1 (10%)	4 (45%)	4 (45%)	9 (100%)	0
43 (100%)	5	7	14	19	39 (91%)	3 (10%)

Overall numbers (43) show that five children (*n* = 5) were rated with a severe medical condition needing a referral immediately (12%). Of these, one 6-month-old child was diagnosed with craniosynostosis and referred for CT scan and craniofacial consultant review, in addition to being developmentally delayed and not sitting unsupported or rolling. One 5-year-old child was diagnosed with severe Autism Spectrum Disorder. The Nurse Practitioner was unable to perform assessment due to challenging behaviour but witnessed difficult behaviour and discussed required referrals with the guardian. One 3-year-old child was diagnosed as developmentally delayed, was not immunised and with a possible foot fracture. One 3-year-old child presented with no immunisations. One 1-year-old child was diagnosed with severe plagiocephaly and gross motor delay. Of note despite the immediacy and severity of the need by children referred for immediate interventions, they were required to wait a further 3 months by the public hospital service before being triaged into the system and waiting an additional 3 months to be seen. This resulted in a delay of 6 months for children with a developmental delay due to, for example, craniosynostosis. These developmental delays require immediate intervention.

Overall, six children (*n* = 6) were rated with moderate medical conditions and needed a referral but could wait for a public clinic (14%). Three of these were dental issues presenting with intermittent pain and required primary teeth extractions. One 16-year-old child was previously diagnosed with scoliosis and ankylosing spondylitis but had never seen a spinal surgeon and needed referral to a spinal specialist, dietician and physiotherapy. The conditions were poorly managed, with the child anxious, having quit school due to bullying and was being home schooled. One 3-year-old child was referred to the children’s assessment team at a local hospital with severe hearing problems. One 8-year-old child had large tonsils and recurrent ear infections. One 2-year-old child was diagnosed with possible autism and developmental delay, poor nutrition and poor oral hygiene.

Overall, 10 children (*n* = 10) were diagnosed with minor medical issues not needing immediate referral (23%). These conditions ranged from chronic dental concerns, poor dental hygiene, viral cough, enlarged tonsils, dental decay, mild speech delay, minor behavioural issues, head lice, possible sleep apnoea, poor school attendance and benign umbilical hernia. Three children (*n* = 3) were listed with medical conditions, but no severity rating attached to them (7%). These conditions were listed as mental health and behavioural concerns, possible additional teeth and one child being very small – low weight and height, both well below the 3rd centile, multiple primary teeth extracted due to decay and blue sclera, possible undiagnosed Osteogenesis imperfecta needs further investigations to confirm or exclude this. Overall, 19 children who attended were in good health and not needing any follow-up (44%). Overall, 91% of children were unsure or not able to show proof of up to date immunisation (*n* = 39) and of these 23% were definitely not immunised as stated by carer, parent or grandparent (*n* = 10). The number of home visits required was high. Overall, 81% of all children required home visits (*n* = 35) due to lack of transport and the impact of COVID-19 restrictions, as well as reasons stated around parent trauma history, agoraphobia and anxiety issues.

Eleven percent of the First Nations children needed medical interventions; there were zero severe conditions needing immediate referral to external health services (*n* = 0), one child with moderate severity condition (*n* = 1) referred to a dentist (2%), and four children (*n* = 4) with minor severity conditions (9%); two (*n* = 2) referred to dentist, one (*n* = 1) referred to ear, nose and throat specialist (wait time 3 years) and one (*n* = 1) referred to General Practitioner (GP) for immunisations. Four children (*n* = 4) rated zero in severity and in general good health, but with concerns for lack of school attendance (9%). None of the First Nations children were able to show proof of immunisation, meaning that all nine (*n* = 9) First Nations children could potentially be non-immunised (100%).

### Social issues

The study identifies five areas for consideration: missed care for all of the children, but a lower rate for First Nations children; lack of access to mainstream services; health care costs as a barrier to service access; poor referral uptake and lack of information on the number of homeless children, particularly First Nations children. The discussion addressed the lower rates of health care problems for First Nations children.

## Discussion

This study found that the Nurse Practitioner-led services provide holistic and assertive care for children living in housing insecurity when they first arise before they become difficult to resolve. Innovative services that directly support First Nations children and their families to navigate health and social systems are needed, and this NP-led clinic addressed many of the gaps in existing health access for children. The Nurse Practitioner was able to identify medical and dental needs at various levels of severity and provide education, advice and appropriate referrals where required. The evidence to date indicates that the model has led to enhanced access to health care and increased uptake of referrals, leading to improved health outcomes for homeless/housing insecure children who through traditional models of health service delivery miss out on urgent acute care and medical interventions. The model has changed how the children receive care and how the community delivers health directly to children in need.

While the numbers of First Nations children referred to the service were low, some comment on the lower numbers of severe conditions, but low rate of immunisation of the First Nations children is required. As noted above, it is well known that First Nations peoples can face many challenges when accessing mainstream services. These include unwelcoming hospital settings, lack of transport, mistrust of mainstream health services and a sense of alienation, and inflexible treatment options (Vallesi *et al.*, 2020). This could be a contributing factor to the lower uptake of First Nations families to the NP clinic compared to non-First Nations children accepting and utilising the service; the NP clinic did not have a specific First Nations focus, staff or anchorage in Aboriginal Community Controlled Health Services, this could have contributed to less uptake. Additionally, historical mistrust of government institutions and a lack of culturally acceptable services and resources mean that parents/carers may only seek help when there is a crisis (DiGiacomo *et al.,* 2017).

Lack of engagement with services emerges as findings in the data, such as the low immunisation rates. Communication between mainstream health services and families is imperative in achieving successful health outcomes. Research has indicated that poor communication from health providers and lack of First Nations staff at health services exacerbate the problem (Durey *et al*., 2011; Isaacs *et al*., 2012). To resolve this, health services need to commit to developing respectful partnerships with local First Nations communities and increase the capacity of services to be more responsive to First Nations People’s requirements (Taylor *et al*., 2013; Taylor and Thompson, 2011). First Nations families may have been unaware or have little knowledge of the NP services and supports, their eligibility or how to access them. Low income and lack of transport are additional barriers to services for carers (DiGiacomo *et al*., 2017). Given the evidence that cultural care is essential to enhance quality of general care for First Nations families and children, it is critical for health services to ensure that cultural care is more consistently evident (Sivertsen *et al*., 2019). There is cultural and research evidence that supports this approach. Adding First Nations health personnel to this health care model would further improve benefits for First Nations family users. First Nations clinicians skilled in clinical yarning would be able to find common ground and develop the interpersonal relationships, facilitate the patient’s health story while interpreting it through a biomedical or scientific lens and use stories and metaphors as tools for patients to help them understand a health issue so a collaborative management approach could be adopted (Lin *et al.*, 2016). Nurse Practitioner care is highly valued by patients when measured using scoring tools (Agosta, 2009), with further potential to improving health outcomes for Aboriginal families.

Kinship care is currently the most common form of care for First Nations children (DiGiacomo *et al.,* 2017). The strong sense of reciprocity that characterises First Nations cultural norms may have played a major part in maintaining the health of these children. Certainly, the research on food security supports this hypotheses, with evidence that families will live together during times of extreme vulnerability in order to ensure children are well cared for, or provide incidental care across household (McCarthy *et al.*, 2018). It is well documented that First Nations families endure overcrowding within their homes, and while this is primarily explained by lack of adequate supply, it is also a result of a strong sense of obligation to extended family members who may be in need (Vallesi *et al.*, 2020). Reciprocity and cultural norms of lending assistance beyond the nuclear family may explain why these children did not have the severe health problems that the non-Indigenous cohort did. However, this explanation requires further research, given the numbers presented here are low.

## Concluding remarks

Homeless service staff informal feedback about the Nurse Practitioner and the service provided was that the nurse has been consistently flexible with the volatile and spontaneous needs of clients (e.g., responding to emergencies, changing appointment times) and has demonstrated that she is extremely knowledgeable with navigating the health system. Without a Nurse Practitioner-led intervention in the homeless service, these children would remain disconnected from health and well-being services including education. Nurse Practitioners are well positioned to provide targeted and in-depth health care to complex families and vulnerable populations who might otherwise have to use the ED or missed care.

This article is foundational as the research it reports and addresses a serious lack of research in the area of childhood outcomes for First Nations children and non-First Nations children living in housing instability. The lack of health service interest and the lack of government focus on the 22% of children in Australian living in housing instability are disturbing, and much more research is required to understand the extent of the deficits in service delivery and its ongoing implication for children across their lifespan.

This proof of concept study underpins the next phase which will include a more systematic exploration of cost-benefit analyses and upscaling the Nurse Practitioner model. More importantly, the next project phases will provide room for the voices of the First Nations families on the service, the venue and the Nurse Practitioner. Phase 2 will utilise the KWY and Kinship Care Organisations to help recruit a First Nations researcher to conduct yarning circles as part of the qualitative data collection methods.

## Limitations and recommendations for future practice

Ideally, a First Nations Nurse Practitioner, RN or the use of First Nations peoples, Health Workers/Practitioners could further mitigate some of the barriers to health access and connections to the service while improving cultural representation in UCWB. Sustainability and funding from state health are needed as well as extended Medicare funding for Nurse Practitioner services expanded to include in-depth and comprehensive child health assessments and referrals, extensive referral support and follow-up. Additionally, it is imperative that Nurse Practitioners in homeless services operate within a culturally safe framework, and the employment of First Nations staff in the service would enhance this.

This study was undertaken in urban South Australia and it may not reflect similar representation of First Nations peoples in other places in Australia. First Nations Peoples consist of many and varied communities, in rural, remote and urban settings, and as such have different cultural traditions, beliefs and health care experiences.

## Terminology

In this study, First Nations Peoples respectfully encompass the diversity of Aboriginal and Torres Strait Islander cultures and identities in Australia. See recommendations for language use by Reconciliation Australia (Reconciliation Australia, 2021).
